# Revolutionizing Neonatal Care: A Comprehensive Assessment of Neuromotor Development in At-Risk Infants Using the Novel Neonatal Infant Motor Assessment Scale (NIMAS) Test Battery

**DOI:** 10.3390/children11040445

**Published:** 2024-04-07

**Authors:** Mustafa Ali Akin, Nilay Comuk Balci, Mert Demirsoz

**Affiliations:** 1 Department of Neonatology, Faculty of Medicine, Ondokuz Mayis University, Samsun 55139, Turkey; mustafaali.akin@omu.edu.tr; 2 Department of Physiotherapy and Rehabilitation, Faculty of Health Sciences, Ondokuz Mayis University, Samsun 55139, Turkey; 3 Department of Biostatistics, Selcuk University, Konya 42130, Turkey; mertdmrsz17@gmail.com

**Keywords:** premature infant, newborn, infant development, risk

## Abstract

We developed a new neonatal neuromotor test battery, the Neonatal Infant Motor Assessment Scale (NIMAS), to perform a detailed neuromotor and holistic assessment of at-risk infants in the neonatal period. Methods: A total of 68 infants (28–41 Gestational weeks) hospitalised in the Neonatal Intensive Care Unit were included in the study. The NIMAS is a scale consisting of Automatic Motor Area, Functional Motor Area and sociodemographic form. The Dubowitz Neurological Examination and the Amiel-Tison Neurological Assessment Tests were also applied to evaluate the construct validity of the test. Results: The mean gestational age at birth was 34.62 ± 3.07 weeks and birth weight was 2305.66 ± 738.95. Fifty-one (75%) of the babies were premature and 17 (25%) were term babies. The KMO value to test the adequacy of the distribution for factor analysis was found to be at a very good level. Barlett’s test result was 2198.389 (*p* < 0.05). The amount of variance obtained as 44.76% in the study was at a sufficient level. The factor loads of the questions in the automatic motor domain dimension varied between 0.523 and 0.694 and the factor loads of the questions in the functional motor domain dimension varied between 0.619 and 0.772. Since Cronbach’s alpha was above 0.70, the reliability was adequate. Inter-rater scale agreement in the automatic motor domain was 81.1%; scale agreement in the functional motor domFain was 92.9%; and the NIMAS total score agreement was 93.4%. These agreements were statistically significant (*p* < 0.05). Total correlation above 0.20 indicates that the item is important for the question. According to the results obtained, total correlation values were between 0.258 and 0.720. Conclusions: The NIMAS is the first test battery to assess the “Functional Motor Area” and this questionnaire, based on the results of the analyses, is a valid, reliable and clinically usable measurement tool for the infant at-risk at the neonatal period.

## 1. Introduction

The early evaluation of at-risk newborn infants in neonatal clinics is crucial for identifying and addressing potential developmental challenges that may arise in this vulnerable population. Assessing the neuromotor competence of newborns is particularly important for timely intervention and support [1]. Infants at risk are identified by the presence of adverse environmental and biological factors that increase their susceptibility to neurodevelopmental disorders and mortality. Factors such as prematurity, perinatal asphyxia, hypoxic-ischemic encephalopathy, periventricular leukomalacia, intraventricular hemorrhage, chronic lung disease, seizures, meningitis, hyperbilirubinemia, twins/triplets, and intrauterine growth restriction contribute to the risk of morbidity and mortality in infants. The incidence of developmental disorders in at-risk infants, especially those born preterm and with low birth weight, is higher compared to their healthy counterparts. With advancements in medical care, there has been a notable increase in the survival rate of high-risk infants, emphasizing the importance of thorough neuromotor evaluations. Also, this population has noticed a notable increase in the occurrence of motor impairments later in life. These impairments encompass a spectrum from developmental coordination disorder to cerebral palsy. Early detection of motor problems becomes imperative for facilitating prompt interventions that can significantly impact the developmental trajectory of these infants [2].

Existing evaluation tests for neurodevelopment in infants, such as the Dubowitz Neurological Examination, the Amiel-Tison Neurological Assessment (ATNA), and the General Movements Assessment, have been widely used in the literature. These assessments play a crucial role in identifying neurological abnormalities and predicting developmental outcomes. The Dubowitz Neurological Examination primarily targets reflexes, muscle tone, and neurological signs, serving as a reliable method for both preterm and term infants. The Amiel-Tison Neurological Assessment (ATNA) distinguishes itself by identifying infants with normal development despite risk factors and those with adverse neurological outcomes, incorporating a brief and easily applicable clinical examination approach. The General Movements Assessment specializes in observing the quality and pattern of spontaneous movements, providing early predictions of neurological abnormalities and developmental outcomes [3,4].

Existing literature reveals a scarcity of test batteries employed during the neonatal period, primarily aimed at assessing infants’ motor and neurological status. While these existing batteries offer insights into infants’ neuromotor levels, they may fall short in differentiating between infants in a clinical context. Recognizing this gap, we embarked on a journey to devise a novel test to garner more comprehensive information.

We posit that neonatal infants are not merely passive entities exhibiting minimal movements and reflexes, but rather, they possess immense potential. These infants are unique individuals characterized by biopsychosocial traits, in addition to motor and reflex development. Newborns utilize movement as a means to direct their gaze and attention towards intriguing visuals and sounds, adjust their positions, express their needs, explore their bodies, and seek comfort. Therefore, an early evaluation of their posture and their ability to control specific movements can provide valuable insights into their overall functionality within their environment. Our primary objective was to create a test battery that would facilitate a more pronounced differentiation of infants during neuromotor assessment, while simultaneously evaluating and emphasizing their social, functional, and additional motor characteristics. In consideration of these aspects, we developed the NIMAS test battery. This test battery encapsulates crucial motor developmental features of infancy, including reflexes, reactions, rotation, posture, asymmetry, functional movements, and functional development. The necessity of such multifaceted neonatal testing lies in its potential to guide early interventions tailored to the specific needs of at-risk infants. By identifying motor, functional, and psychosocial challenges early on, healthcare professionals can implement targeted interventions, potentially mitigating developmental risks and enhancing overall neonatal well-being. The introduction of a comprehensive tool, specifically designed to assess the “Functional Motor Area”, presents a unique opportunity for a more holistic and three-dimensional evaluation of newborns [1,5].

This study aims to investigate the validity and reliability of the NIMAS as a measurement method for assessing the neuromotor competence of at-risk newborns. This investigation not only seeks to validate and establish the reliability of the NIMAS but also highlights its modern aspects, encompassing a broad evaluation of body movements, behaviour, consciousness, facial movement, social behaviour, posture, and asymmetry.

## 2. Materials and Methods

This study investigated the neuromotor development of a cohort of newborn at-risk infants admitted to the Neonatal Intensive Care Unit (NICU) at the Department of Neonatology, Ondokuz Mayis University Hospital, from April 2022 to April 2023. The study adhered to the ethical standards outlined in the 1964 Declaration of Helsinki and its subsequent amendments. Approval for the study was obtained from the Ethics Committee of Ondokuz Mayis University Ethics Board (2022/117; 16 March 2022), and written informed consent was obtained from all participants after providing informed parental consent.

The sample size, calculated using G*Power 3 (version 3.0.10, Franz Faul, Universität Kiel, Kiel, Germany) [6], comprised 65 at-risk newborn infants for the test–retest reliability analysis, with 98.8% power. At-risk infants included those with early preterm birth (˂32 weeks), moderately preterm birth (32–34 weeks), multiple births, hypoxic-ischemic encephalopathy, bronchopulmonary dysplasia, intraventricular haemorrhage, antenatal haemorrhage, large for gestational age, and periventricular leukomalacia between 26–44 postconceptional weeks. Infants were excluded if they had known progressive neurological disorders (such as early-onset myotonic dystrophy, genetic refractory epileptic encephalopathy, refractory focal epilepsy, or structural West syndrome), congenital anomalies, musculoskeletal disorders, cyanotic congenital heart disease, or mechanical dependency.

The study followed an observational, longitudinal design. The Neonatal Infant Motor Assessment Scale (NIMAS, NCB-MAA-MD, Samsun, Turkey) was performed on newborn infants in the NICU by senior physicians (NCB, MAA) with over 5 years of experience in assessing high-risk newborn infants. Infant baseline characteristics, including sex, birth weight, gestational age (GA), GA range at birth, small for gestational age (SGA) status, mode of delivery, and Apgar scores at 1 and 5 min, were collected from hospital charts and recorded in the patients’ sociodemographic and medical forms.

Brain lesions were defined based on findings from cranial ultrasound (cUS) according to the local clinical imaging protocol. Severe brain lesions were characterized by grade III intraventricular haemorrhage, parenchymal hemorrhagic venous infarction, post-hemorrhagic ventricular dilation, focal cerebellar haemorrhage, cystic periventricular leukomalacia, more than 6 punctate white matter lesions, or brain malformations [7,8].

Interrater reliability was assessed by two assessors evaluating the same patient using the NIMAS, on the same or consecutive day. Concurrent validity was examined using the Dubowitz Neurological Examination and Amiel-Tison Neurological Examination tests on the same day. The Dubowitz and ATNA tests were conducted by an assessor who was intentionally kept uninformed about the study’s objectives. The baseline assessment, including all tests and a rest interval between tests, took approximately 40–45 min to ensure the infant was at a comfortable behavioural level. Assessors were blinded to each other’s scores in all cases.

### 2.1. Neonatal Infant Motor Assessment Scale (NIMAS)

The NIMAS, developed as a test battery for evaluating the neuromotor domain in infants, was designed for biopsychosocial assessment during the neonatal period (26–44 postconceptional weeks). Comprising a total of 36 items, the scale was divided into three subdomains: Sociodemographic Form, Automatic Motor Area (AMA), and Functional Motor Area (FMA).

**Figure 1:**
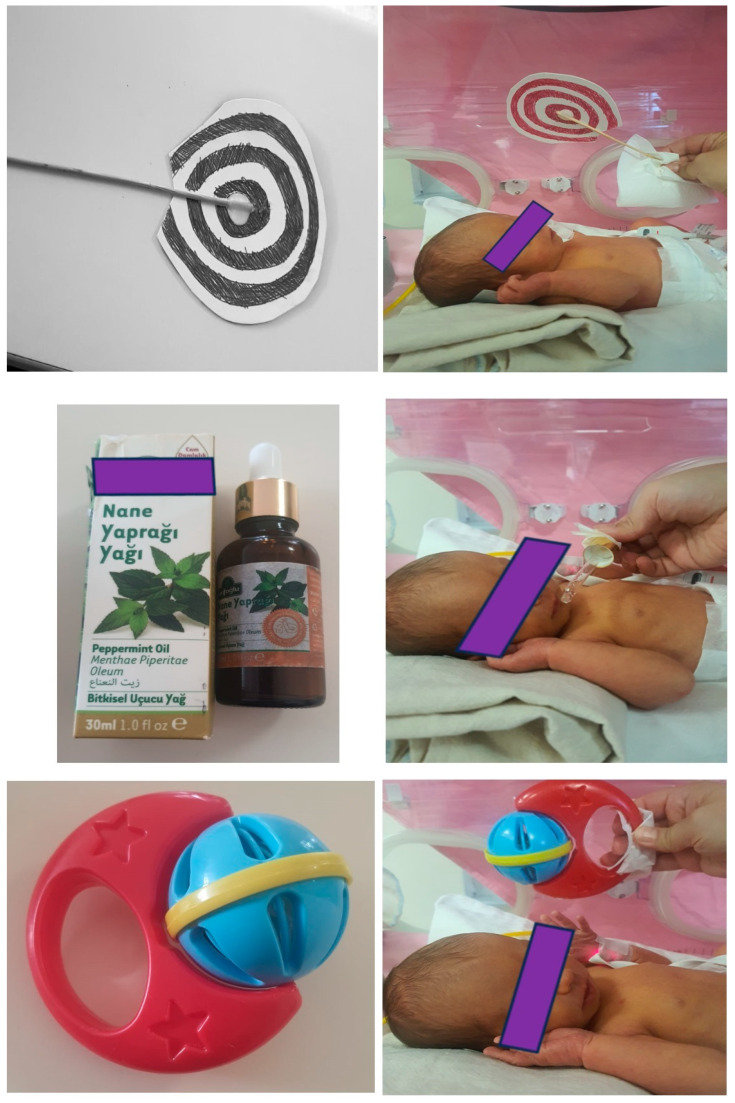
Test equipment of the NIMAS.

The Sociodemographic Form was an informative scale that does not contribute to scoring but contains birth and medical information related to the infant. AMA encompassed reflexes, plantar response, tone, cranial nerve functions, posture, and asymmetry. FMA evaluated subdomains such as body movement, rotation, turning, spontaneous finger movement, bringing hands to the midline, and flexing legs to the abdomen. It also included the assessment of facial expressions, consciousness, and social behavior. Ach question was scored as 1, 2, or 3, where higher scores indicated a better level. The scoring ranged from a minimum of 36 to a maximum of 108 points. The assessment took approximately 20–25 min. Required test equipment included a visual tracking card, rattle, and peppermint oil (Figure 1). When evaluating infants in the neonatal intensive care unit (NICU), the assessment should be conducted when the infant was medically stable, approximately 30 min to 1 h after feeding, calm, and with minimal clothing.

### 2.2. The Dubowitz Neurological Assessment of the Preterm and Full-Term Infant-Dubowitz

The Dubowitz Scale for Neurological Assessment of Preterm and Full-term Infants [9] is a standardized tool applicable to both preterm and full-term infants. Its item validity and reliability have been well-established, making it a frequent choice for neurological examinations in newborns [10,11]. Comprising 34 items and 6 subgroups, including tonus, tonus pattern, reflexes, movement, abnormal findings, orientation, and behaviour, the Dubowitz assesses the neuromotor functions of infants [10].

Notably, it offers convenience in the Neonatal Intensive Care Unit (NICU), with an application time of 10 to 15 min. Its user-friendly format includes simple instructions and drawings depicting infant movements. The Dubowitz Infant Neurologic Assessment Scale calculates a raw score, assigning each item a value between 1 and 5 points. Additionally, an alternative combined optimal score calculation method was developed [10]. This method addresses concerns that the raw score calculation might not precisely determine the neuromotor status of premature infants based on gestational weeks, as infants may not exhibit some responses according to their gestational age [9].

In this alternative method, an optimality score is assigned individually for each item. An infant receives 1 point if their movement aligns with the gestational week’s expectations and 0 points if it does not. The total combined optimal score ranges from a maximum of 34 to a minimum of 0 points. For babies born at term, a combined optimal score ≥ 30.5 is considered the cut-off point [11].

### 2.3. Amiel-Tison Neurological Assessment

Following birth, the infant was transferred to the neonatal intensive care unit for careful observation and optimal diagnostic and treatment conditions. The Amiel-Tison test, conducted by the neonatal physiotherapist when the infant reached stability, utilized a non-numeric scoring system based on emerging symptoms and findings.

Throughout the assessment, the neonatal physiotherapist evaluated the infant’s cranial characteristics, alertness, behaviour, spontaneous activity, active and passive tone in the infant’s trunk, wakefulness, primary reflexes, and extremity tone. Test results were determined by the severity of the evaluated neuromotor parameters, with evaluation parameters scored as follows: “0” for a typical result within the normal range, “1” for a moderately abnormal result, and “2” for a severely abnormal result.

If infants scored 0 points across all parameters, they were given the label “central nervous system function is optimal”. If infants received a score of 1 or 2 in some evaluation parameters, the result indicated “mild, moderate, or severe exposure in the central nervous system”. For premature infants reaching corrected term age, the designation of “central nervous system function is optimal” was assigned if they scored 0 in all parameters. Alternatively, they received one of the results indicating “mildly, moderately, or severely affected in the central nervous system” if they scored 1 or 2 in some evaluation parameters.

During assessments, infants undergoing mechanical ventilation and intubation were categorized as having “severe exposure”. Evaluations were preferably conducted when the infant was awake, calm, and not hungry [12,13,14].

### 2.4. Statistical Analysis

In the realm of explanatory factor analysis, factors formed by observed variables were identified. These factors represent hypothetical constructs [15]. To assess the data’s suitability for factor analysis, the correlation matrix was examined. It was determined that coefficients in the correlation matrix should not exceed 0.30 for compatibility with the underlying factor structure [16]. The Bartlett test of sphericity was employed for the statistical testing of correlations between variables in the data matrix [17]. This test verified whether the matrix formed among the questions was a unit matrix. Additionally, the Kaiser-Meyer-Olkin (KMO) criterion, derived from correlation and partial correlation coefficients, assessed the data’s suitability for factor analysis. A KMO value greater than 0.5 was considered adequate [18].

In this study, the basic component method was used to obtain factors. The number of factors selected took into account eigenvalues larger than one. Factor rotation was applied using the varimax method to enhance the clarity of variables contributing to each common factor. Observer agreement was assessed using an intraclass correlation coefficient (ICC), with a reliability threshold set at 0.70 or higher [19]. A value of 0.20 or higher for the item-total correlation coefficient indicated the item’s consistent alignment with the overall scale [20]. Prior to scale development and structural validity testing, explanatory factor analysis is typically applied. Inter-rater comparisons were conducted using the Wilcoxon test.

Confirmatory factor analysis, on the other hand, is employed to validate the structure obtained from explanatory factor analysis or a theoretical factor structure [21]. While explanatory factor analysis determines an appropriate number of factors based on the data matrix, confirmatory factor analysis presumes a known number of factors. IBM SPSS Statistics for Windows (Version 25.0) and Amos (Version 24.0) were utilized for confirmatory factor analysis in this study.

Data evaluation using IBM SPSS Statistics Standard Concurrent User V 26 [22] included descriptive statistics such as unit count (n), percentage (%), mean (M), standard deviation (SD), median (Md), and minimum (min) and maximum (max) values. The normal distribution of numerical variables was assessed using the Shapiro-Wilk normality test. Mann-Whitney U Test was used for two-group comparisons, Kruskal-Wallis H Test for comparisons involving more than two categorical variables, and Bonferroni test for multiple comparisons if the variance analysis result was significant. Relationships between numerical variables were evaluated using the Spearman correlation coefficient, with statistical significance set at *p* < 0.05

## 3. Results

Upon reviewing Table 1, it was evident that among the 68 infants participating in the study, the median gestational age at birth was 35 weeks, with the earliest birth recorded at 28 weeks and the latest at 41 weeks. The median birth weight for these infants was 2330 g, with weights ranging from 2100 g. Notably, 51 infants (75%) were classified as premature, while 17 infants (25%) were not. It is observed that among the 68 participating infants in the study, the median age of the mothers was 28 years, with the youngest mother being 18 years old and the oldest being 46 years old. The median number of pregnancies for the mothers is found to be 2.

As indicated in Table 2, the average Dubowitz score was 17.24 ± 6.11 points, ranging from a minimum score of 4 to a maximum of 33. The average Amiel-Tison score was 15.50 ± 9.85 points, with scores ranging from 0 to 41. In terms of Amiel-Tison classification, 16 individuals (23.5%) were categorized as normal, 29 individuals (42.6%) as minor, 17 individuals (25%) as moderate, and 6 individuals (8.8%) as severe.

In Table 3, the adequacy test for the factor analysis distribution revealed a highly favorable Kaiser-Meyer-Olkin (KMO) value. The Bartlett test resulted in 2198.389 (*p* < 0.05), signifying a robust statistical significance. The obtained variance in the research, standing at 44.76%, can be considered sufficient. According to the table, the factor loadings for questions in the automatic motor dimension range from 0.523 to 0.694, while those in the functional motor dimension range from 0.619 to 0.772, indicating notable variability. With a Cronbach Alpha exceeding 0.70, the reliability was deemed satisfactory, affirming that the NIMAS scale adequately measured the underlying sub-attributes of the two dimensions. Consequently, the survey crafted based on these results stands as a reliable measurement tool.

Inter-rater agreement was found to be 81.1% for the automatic motor dimension, 92.9% for the functional motor dimension, and 93.4% for the total NIMAS score as in Table 4. These agreements were statistically significant (*p* < 0.05). A total correlation exceeding 0.20 indicates the significance of the item for the question. The total correlation values obtained ranged from 0.258 to 0.720. Based on these results, the questionnaire appears to be a valid measurement tool. Furthermore, items 1, 3, 4, 5, 6, 10, 25, 26, 27, and 33 exhibited statistically lower scores for the first evaluator in both the functional motor and total NIMAS scores (*p* < 0.05).

Upon scrutinizing Table 5, the χ^2^/df, RMSEA, SRMR, IFI, TLI, CFI, and GFI were employed within the scope of Discriminant Function Analysis (DFA) to assess the factor validity of the models. RMSEA is an index least affected by sample size; however, criteria for RMSEA fit index vary in different literature. Generally, a cutoff value close to 0.06 or 0.08 is deemed acceptable. An IFI, TLI, CFI, and GFI exceeding 0.90 are considered evidence of sufficient model fit. In this study, criteria were set as RMSEA ≤ 0.05, IFI, TLI, CFI ≥ 0.90, and GFI ≥ 0.85 for acceptability. The model obtained for the NIMAS scale (χ^2^ = 1188.329, df = 575) comprises two dimensions. The fit indices for this model demonstrate that it is acceptably well-fitted.

**Figure 2:**
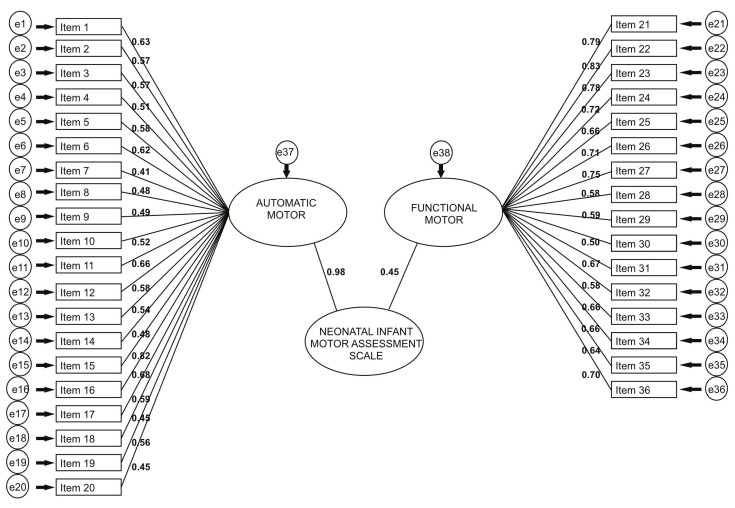
Confirmatory factor analysis model of the NIMAS.

The NIMAS scale, consisting of 36 items and two dimensions, underwent confirmatory factor analysis. The model is visually presented in Figure 2. The path coefficients for each question on the 36-item scale are statistically significant (*p* < 0.05), affirming that the Automatic Motor dimension encompasses questions 1–20, while the Functional Motor dimension comprises questions 21–36. All sub-dimensions exert a highly statistically significant impact on the questions. Additionally, the path coefficients for both the Automatic Motor and Functional Motor dimensions on the NIMAS scale are individually statistically significant (*p* < 0.05).

The average score for the Automatic Motor dimension of the scale was calculated as 2.34 ± 0.27 points, while the mean total score of the scale was found to be 2.25 ± 0.26 points. The total score of the scale was obtained by dividing the total sum of scores by the number of questions, and there were no reverse-scored items in the scale. The scale ranged from a minimum score of 1 point to a maximum score of 3 points. A statistically significant positive relationship was observed between the dimensions of the NIMAS scale (*p* < 0.05) (Table 6).

Table 7 illustrates a statistically significant positive correlation between the Dubowitz scores and the NIMAS, as well as its dimensions (*p* < 0.05). Similarly, there was a statistically significant positive correlation between the Amiel-Tison scores and the NIMAS, including its dimensions (*p* < 0.05).

Table 8 demonstrates a statistically significant positive correlation between automatic motor skills and variables such as gestational weeks, birth weight, corrected age, Apgar score at 5 min, and gravida. (*p* < 0.05). Additionally, a positive and statistically significant relationship was found between functional motor skills and the Apgar score at 5 min (*p* < 0.05). The NIMAS showed a positive statistically significant correlation with corrected age, and Apgar score at 5 min. (*p* < 0.05).

As shown in Table 9, the average scores of the NIMAS scale were statistically lower in infants fed with OG (*p* < 0.05). On the other hand, the average scores of the NIMAS scale were statistically higher in individuals with a normal Amiel-Tison range and normal cUS (*p* < 0.05).

## 4. Discussion

The early assessment of at-risk newborns in neonatal clinics is crucial for their development, requiring the utilization of valid and reliable scales. This study aimed to determine the validity and reliability of the Neonatal Infant Motor Assessment Scale (NIMAS) as a measurement method for newborns aged 26–44 weeks postconceptional ages. The findings highlight NIMAS as a valid and discriminating measure for assessing the neuromotor competence of at-risk newborns within the 26–44 weeks age range.

Various neuromotor and neurobehavioral scales exist for the assessment of at-risk infants based on their intended use. These scales are employed for the detection of disorders related to the central nervous system function, examination of the relationship between neuromotor and behavioural functions, determination of the risk of potential complications in the future, monitoring the infant’s development, and assessment of the effectiveness of interventions applied [23]. The incidence of developmental disorders is higher in at-risk infants, emphasizing the need for early detection of motor problems. The rise in medical care facilities has increased the survival rate of high-risk infants born preterm or with low birth weight. Consequently, evaluating the neuromotor aspects of high-risk infants becomes imperative. Common evaluation tests like the Dubowitz Neurological Examination, the Amiel-Tison Neurological Assessment, and the General Movements Assessment are extensively used in the literature in the neonatal period [23]. However, the NIMAS has some similarities and also differences from other assessments that have evaluated both automatic and functional aspects of the neuromotor development in a holistic way which is also suitable for the biopsychosocial aspects of the human being.

The Dubowitz Neurological Examination, a reliable method for both preterm and term infants, helps in the early identification of markers for brain injury and response to treatment. Early neurological examination may serve as a marker for brain injury after hypoxia ischaemia as well as a response to treatment. Detailed neurological examination in term and preterm infants using the Dubowitz neonatal examination after the second postnatal week was predictive of neurodevelopmental outcome. The lowest scores were associated with severe white matter injury as seen on magnetic resonance imaging (MRI), hence, optimality scores gave prognostic information on the severity of functional motor outcome in this population [10,11] We also used the Dubowitz Neurological Examination and found a positive medium correlation with the NIMAS.

The Amiel Tison Neurologic Assessment (ATNA) proved to be useful in recognizing infants who have normal development despite risk factors, and those who have adverse neurological outcomes and delayed developmental performance [24]. We showed good agreement between the ATNA and the NIMAS results as well as demonstrating the value of this kind of examination of aetiological orientation and of timing of the lesion. The assessment is not difficult or lengthy and can be incorporated into the routine examination of neonates. The value of ultrasound in diagnosing CNS pathology and correlations between neurological signs and specific ultrasound abnormalities have been confirmed in many studies and the correlation of specific clinical patterns with magnetic resonance imaging findings has also been analyzed. In our study, according to ATNA results, there were 16 (%23.5) normal, 29 (%42.6) infants with mild neurologic signs, 17 (%25) infants with Moderate neurologic signs, 6 (%8.8) infants with Severe neurologic signs. In our study, according to the results, there were 57 (%83.8) infants with Normal cranial Ultrasound and 11 (%16.2) infants with abnormal cranial Ultrasound. These results were in correlation with the NIMAS, Dubowitz and Amiel Tison-Scores.

The existing tests in the literature are utilized to assess the motor and neurological condition of infants. Also, the availability of test batteries during the neonatal period is limited. While the current batteries offer insights into infants’ neuromotor levels, they may lack the specificity needed to differentiate between infants in clinical settings. Recognizing this gap, we embarked on developing a new test to gather more comprehensive information. We believe that infants in the neonatal phase possess untapped potential beyond a few movements. They have biopsychosocial characteristics. Typically, motor evaluations in the literature follow the neuromaturational model, which presupposes a consistent pace and sequence in motor development, aligning motor skill acquisition with the central nervous system’s hierarchical structure.

Nevertheless, there have been advancements in our assessment tool the NIMAS, which embraces the dynamic systems theory. This theory views motor skill development as a product of various interacting subsystems and emphasizes the task context’s influence. Assessments grounded in dynamic systems theory gauge functional capacity while considering environmental factors to optimize infants’ performance. Our primary aim was to create a test battery that not only enhances the differentiation of infants during neuromotor evaluations but also evaluates their social, functional, and additional motor attributes. This battery encompasses essential functional motor developmental aspects of infancy.

In the literature, these tests provide information about cranial nerve functions, reflexes, reactions, tonus, spontaneous movements and motor development stages of the infant. Although the NIMAS evaluates these areas of the infant, unlike these tests, it includes a history sheet including an intensive medical condition and reflex history of the infant, response to the sole of the foot, tonus assessment for all parts of the body, visual tracking in cranial nerve functions, ability to focus on the midline in vision and response to smell, posture both in the supine position and in the sitting position, The presence of asymmetry for all parts of the body, evaluation of rotation movements for all parts of the body, limb responses to rotation, spontaneous finger movements, facial condition, state of consciousness, social responses to touch and speech, ability to bring hands to the midline, ability to pull knees to the abdomen. We can say that the presence of all these assessment areas in different directions in the NIMAS supports the biopsychosocial evaluation of infants and the dynamic systems theory.

The importance of such comprehensive neonatal testing lies in its potential to inform early interventions tailored to each infant’s specific needs. Identifying motor, functional, and psychosocial challenges early on allows healthcare professionals to implement targeted interventions, potentially mitigating developmental risks and improving overall neonatal well-being. Introducing a comprehensive tool, particularly focused on assessing the “Functional Motor Area”, presents a unique opportunity for a holistic and three-dimensional evaluation of newborns.

Our study had some limitations. While the number of infants in our study was sufficient to complete the study, the subgroups consisted of heterogeneous groups of preterm and term infants. Further studies are needed to establish the distribution of scores in preterm infants and in term infants. Second, we collected data from one center, limiting the possibility of generalizing our findings from a single study center to a large cohort of infants. The practicality of assessment tools is evaluated based on their validity and reliability. Certain tools like General Movements and the Bayley Assessment necessitate standardized training and can be expensive, but this investment can enhance the reliability and validity of the assessments [4]. It is envisaged to conduct free practical training through the NIMAS test’s dedicated website in the future, explaining how each test item is administered. This way, every professional administering the test will have detailed knowledge of how to score each item. We believe that this will enhance the validity and reliability of the test regarding its administration. Also, confident clinicians may prefer a readily accessible tool requiring minimal training. The NIMAS offers the advantage of easy administration in clinical settings, making it more feasible for physicians to use in clinics due to its streamlined process and time efficiency during assessments.

## 5. Conclusions

The NIMAS is the first test battery to assess the “Functional Motor Area”, and according to the results of the analyses, this questionnaire is a valid, reliable and clinically usable measurement tool. The strengths and modern aspects of the NIMAS are that it provides a holistic evaluation of the infant, a three-dimensional evaluation of body movements, an evaluation of behaviour, consciousness, facial movement and social behaviour, an evaluation of posture and asymmetry in many areas and is a test that manipulates the infant less. The data are important for studies or trials of neonatal treatments in which neurological examination will be used to select for neuroprotective studies, assess progress of disease and predict outcomes. Further studies are needed to establish the distribution of scores in preterm infants and in term infants with suboptimal but less extensive abnormality on cranial ultrasonograms and to determine the diagnostic and prognostic value of the examination for the infant who falls in this category. Further studies are also needed to establish whether the lower scores may relate to the minor motor signs frequently found in preterm infants or high-risk infants on longer-term follow-up.

## Figures and Tables

**Table 1:** Descriptive statistics of characteristics of the infants (n *=* 68).

	Statistics
Gestational weeks, (week)	
X ± SD	34.62 ± 3.07
M (min–max)	35 (28–41)
Birth weight, (grams)	
X ± SD	2305.66 ± 738.95
M (min–max)	2330 (980–3990)
Corrected age, (weeks)	
X ± SD	36.50 ± 2.83
M (min–max)	36 (29–44)
Premature, n (%)	
Yes	51 (%75)
No	17 (%25)
Apgar 1 min. (point)	
X ± SD	6.13 ± 1.39
M (min–max)	6 (1–9)
Apgar 5 min. (point)	
X ± SD	7.25 ± 2.09
M (min–max)	8 (1–9)
Gender, n (%)	
Girls	21 (%30.9)
Boys	47 (%69.1)
cUS, n (%)	
Normal	57 (%83.8)
Abnormal	11 (%16.2)
Maternal age, (years)	
X ± SD	28.21 ± 5.53
M (min–max)	28 (18–46)
Gravida	
X ± SD	2.26 ± 1.22
M (min–max)	2 (1–5)
Feeding type, n (%)	
Bottle	49 (%72.1)
OG	19 (%27.9)
Diet type, n (%)	
Breast milk	37 (%54.4)
Formula	21 (%30.9)
Mixed	10 (%14.7)

cUS: Cranial Ultrasound, OG: Orogastric tube.

**Table 2:** Descriptive statistics of the Dubowitz and Amiel-Tison scores (n *=* 68).

	Statistics
Dubowitz score, (point)	
X ± SD	17.24 ± 6.11
M (min–max)	18 (4–33)
Amiel Tison score, (point)	
X ± SD	15.50 ± 9.85
M (min–max)	15.5 (0–41)
Amiel Tison Range, n (%)	
Normal	16 (%23.5)
Minor Neurological Signs	29 (%42.6)
Moderate Neurological Signs	17 (%25)
Severe Neurological Signs	6 (%8.8)

**Table 3:** The NIMAS reliability results.

Factor	Item Number	Factor Loads		Explained Variance %	Cronbach Alpha
		1	2		
Automatic Motor	1		0.618	21.03	0.902
	2		0.588		
	3		0.554		
	4		0.526		
	5		0.523		
	6		0.542		
	7		0.545		
	8		0.585		
	9		0.576		
	10		0.603		
	11		0.670		
	12		0.559		
	13		0.526		
	14		0.549		
	15		0.694		
	16		0.682		
	17		0.562		
	18		0.649		
	19		0.693		
	20		0.642		
Functional Motor	21	0.758		23.73	0.930
	22	0.772			
	23	0.726			
	24	0.757			
	25	0.675			
	26	0.672			
	27	0.654			
	28	0.685			
	29	0.679			
	30	0.711			
	31	0.722			
	32	0.651			
	33	0.717			
	34	0.699			
	35	0.619			
	36	0.709			
Scale				44.76	0.920
*KMO =* 0.697 *Df =* 630 *χ*^2^ *=* 2198.389 *p* < 0.001					

*KMO:* Kaiser–Meyer–Olkin test; *Df:* Degrees of freedom.

**Table 4:** The NIMAS validity results.

	ItemNo	Firs Test	Second Test	Test (*p*)	Total Correlation	ICC (%95 GA)
Automatic Motor	1	2.35 ± 0.57	2.53 ± 0.59	***Z =* −3.000 *p =* 0.003**	0.445	**0.815 (0.700–0.886)**
	2	2.40 ± 0.58	2.41 ± 0.67	*Z =* −0.728 *p =* 0.467	0.457	**0.780 (0.641–0.865)**
	3	2.21 ± 0.70	2.35 ± 0.69	***Z =* −2.673 *p =* 0.008**	0.414	**0.893 (0.826–0.934)**
	4	2.86 ± 0.46	2.72 ± 0.69	***Z =* −2.000 *p =* 0.046**	0.504	**0.934 (0.892–0.959)**
	5	1.96 ± 0.82	2.22 ± 0.71	***Z =* −3.819 *p* < 0.001**	0.402	**0.877 (0.800–0.924)**
	6	1.96 ± 0.74	2.09 ± 0.64	***Z =* −2.324 *p =* 0.020**	0.525	**0.880 (0.805–0.926)**
	7	2.90 ± 0.39	2.87 ± 0.45	*Z =* −0.816 *p =* 0.414	0.397	**0.859 (0.772–0.913)**
	8	2.91 ± 0.38	2.88 ± 0.41	*Z =* −1.000 *p =* 0.317	0.460	**0.894 (0.828–0.934)**
	9	1.91 ± 0.79	1.93 ± 0.74	*Z =* −0.258 *p =* 0.796	0.258	**0.894 (0.828–0.935)**
	10	1.58 ± 0.74	1.82 ± 0.75	***Z =* −3.286 *p =* 0.001**	0.293	**0.784 (0.648–0.867)**
	11	1.90 ± 0.65	1.87 ± 0.69	*Z =* 0.001 *p =* 0.999	0.551	**0.903 (0.842–0.940)**
	12	1.24 ± 0.61	1.24 ± 0.63	*Z =* −0.276 *p =* 0.783	0.457	**0.873 (0.794–0.922)**
	13	2.07 ± 0.61	2.01 ± 0.59	*Z =* −0.853 *p =* 0.394	0.526	**0.704 (0.520–0.817)**
	14	2.76 ± 0.58	2.53 ± 0.72	*Z =* −2.009 *p =* 0.064	0.294	**0.702 (0.517–0.816)**
	15	2.12 ± 0.56	2.15 ± 0.55	*Z =* −1.414 *p =* 0.157	0.710	**0.976 (0.961–0.985)**
	16	1.90 ± 0.67	1.91 ± 0.62	*Z =* −0.333 *p =* 0.739	0.485	**0.912 (0.858–0.946)**
	17	2.69 ± 0.60	2.75 ± 0.58	*Z =* −1.414 *p =* 0.157	0.570	**0.911 (0.855–0.945)**
	18	2.97 ± 0.24	3.00 ± 0.00	*Z =* −1.000 *p =* 0.317	0.279	**0.889 (0.820–0.931)**
	19	3.00 ± 0.00	3.00 ± 0.00	*Z =* 0.001 *p =* 0.999	0.348	**0.999 (0.999–0.999)**
	20	3.00 ± 0.00	3.00 ± 0.00	*Z =* 0.001 *p =* 0.999	0.368	**0.999 (0.999–0.999)**
**Total**		2.34 ± 0.27	2.36 ± 0.27	*Z =* −1.224 *p =* 0.221	-	**0.811 (0.693–0.884)**
Functional Motor	21	2.13 ± 0.46	2.13 ± 0.49	*Z =* −0.707 *p =* 0.480	0.618	**0.912 (0.858–0.946)**
	22	2.34 ± 0.77	2.34 ± 0.80	*Z =* 0.001 *p =* 0.999	0.720	**0.962 (0.939–0.977)**
	23	1.85 ± 0.55	1.88 ± 0.50	*Z =* −0.632 *p =* 0.527	0.597	**0.848 (0.753–0.906)**
	24	1.88 ± 0.76	1.94 ± 0.62	*Z =* −0.853 *p =* 0.394	0.488	**0.798 (0.673–0.876)**
	25	1.19 ± 0.47	1.62 ± 0.62	***Z =* −4.393 *p* < 0.001**	0.426	**0.485 (0.166–0.682)**
	26	1.31 ± 0.55	1.65 ± 0.69	***Z =* −3.874 *p* < 0.001**	0.570	**0.646 (0.426–0.782)**
	27	1.06 ± 0.24	1.49 ± 0.59	***Z =* −4.874 *p* < 0.001**	0.594	**0.480 (0.157–0.679)**
	28	2.72 ± 0.64	2.79 ± 0.59	*Z =* −1.508 *p =* 0.132	0.396	**0.883 (0.811–0.928)**
	29	2.88 ± 0.44	2.88 ± 0.44	*Z =* 0.001 *p =* 0.999	0.443	**0.819 (0.707–0.888)**
	30	2.91 ± 0.42	2.82 ± 0.62	*Z =* 0.001 *p =* 0.999	0.322	**0.832 (0.726–0.897)**
	31	2.76 ± 0.43	2.75 ± 0.44	*Z =* −1.000 *p =* 0.317	0.564	**0.98 (0.967–0.988)**
	32	2.88 ± 0.32	2.93 ± 0.26	*Z =* −1.732 *p =* 0.083	0.521	**0.860 (0.773–0.914)**
	33	2.28 ± 0.69	2.15 ± 0.72	***Z =* −2.714 *p =* 0.007**	0.584	**0.920 (0.870–0.951)**
	34	2.38 ± 0.65	2.35 ± 0.64	*Z =* −0.500 *p =* 0.617	0.625	**0.833 (0.729–0.897)**
	35	2.18 ± 0.98	2.28 ± 0.94	*Z =* −1.443 *p =* 0.149	0.571	**0.901 (0.839–0.939)**
	36	1.93 ± 0.72	1.91 ± 0.69	*Z =* −0.447 *p =* 0.655	0.513	**0.961 (0.936–0.976)**
**Total**		2.17 ± 0.30	2.24 ± 0.33	***Z =* −3.850 *p* < 0.001**	-	**0.929 (0.885–0.956)**
**General**		2.25 ± 0.26	2.30 ± 0.27	***Z =* −3.311 *p =* 0.001**	-	**0.934 (0.893–0.959)**

Intra-Class Correlation Coefficient (ICC); Wilcoxon Test (Z; Summary statistics are given as mean ± standard deviation. Sections highlighted in bold are statistically significant (*p* < 0.05).

**Table 5:** Statistical values related to the fit of the NIMAS scale model.

Scale	(χ^2^/df)	RMSEA	SRMR	IFI	CFI	GFI	TLI
Model	2.067	0.076	0.073	0.914	0.906	0.851	0.908

χ^2^/df: Ki-square/degrees of freedom; RMSEA: Root Mean Square Error of Approximation; SRMR: Standardized Root Mean Square Residual; IFI: Incremental Fit Index; CFI: Comparative Fit Index; GFI: Goodness of Fit Index; TLI: Tucker Lewis Index.

**Table 6:** Descriptive statistics of the NIMAS.

	X ± SD	M (min–max)	Automatic Motor	Functional Motor
Automatic Motor	2.34 ± 0.27	2.30 (1–3)	**1**	
Functional Motor	2.17 ± 0.30	2.19 (1–3)	***r =* 0.593 *p* < 0.001**	**1**
NIMAS	2.25 ± 0.26	2.23 (1–3)	***r =* 0.881 *p* < 0.001**	***r =* 0.904 *p* < 0.001**

*r*: Spearman Correlation Coefficient; Summary statistics are given as mean ± standard deviation and Median (minimum, maximum) values. Sections highlighted in bold are statistically significant (*p* < 0.05).

**Table 7:** Relationships between the Dubowitz and Amiel-Tison scores and the NIMAS and its dimensions.

	Automatic Motor	Functional Motor	NIMAS
Dubowitz score	***r =* 0.745 *p =* 0.001**	***r =* 0.520 *p =* 0.001**	***r =* 0.717 *p =* 0.001**
Amiel tison score	***r =* −0.821 *p =* 0.001**	***r =* −0.633 *p =* 0.001**	***r =* −0.811 *p =* 0.001**

*r*: Spearman Correlation Coefficient; Summary statistics are given as mean ± standard deviation and Median (minimum, maximum) values. Sections highlighted in bold are statistically significant (*p* < 0.05).

**Table 8:** Relationships between demographic characteristics and the NIMAS and its dimensions.

	Automatic Motor	Functional Motor	NIMAS
Gestational week	***r =* 0.323 *p =* 0.007**	*r =* 0.112 *p =* 0.364	*r =* 0.197 *p =* 0.107
Birth weight	***r =* 0.325 *p =* 0.007**	*r =* 0.102 *p =* 0.406	*r =* 0.203 *p =* 0.096
Corrected age	***r =* 0.360 *p =* 0.003**	*r =* 0.122 *p =* 0.323	***r =* 0.242 *p =* 0.047**
Apgar 1 min.	*r =* 0.180 *p =* 0.141	*r =* 0.204 *p =* 0.095	*r =* 0.211 *p =* 0.084
Apgar 5 min.	***r =* 0.278 *p =* 0.022**	***r =* 0.261 *p =* 0.032**	***r =* 0.299 *p =* 0.013**
Maternal age, (year)	*r =* −0.112 *p =* 0.361	*r =* −0.015 *p =* 0.901	*r =* −0.075 *p =* 0.544
Gravida	***r =* 0.248 *p =* 0.041**	*r =* 0.091 *p =* 0.462	*r =* 0.208 *p =* 0.088

*r*: Spearman Correlation Coefficient; Summary statistics are given as mean ± standard deviation and Median (minimum, maximum) values. Sections highlighted in bold are statistically significant (*p* < 0.05).

**Table 9:** Comparison of the NIMAS scale based on demographic characteristics.

	NIMAS	Test (*p*)
**Premature**		*Z =* −0.730 *p =* 0.466
Yes	2.23 ± 0.26	
No	2.31 ± 0.23	
**Gender**		*Z =* −0.604 *p =* 0.546
Girl	2.28 ± 0.28	
Boy	2.24 ± 0.25	
**cUS**		***Z =* −2.049 *p =* 0.040**
Normal	2.31 ± 0.25	
Abnormal	2.09 ± 0.27	
**Feeding Type**		***Z =* −3.500 *p* < 0.001**
Bottle	2.32 ± 0.23	
OG	2.08 ± 0.24	
**Diet Type**		*H =* 0.664 *p =* 0.718
Breast milk	2.25 ± 0.26	
Formula	2.23 ± 0.26	
Mixed	2.29 ± 0.25	
**Amiel tison range**		***H =* 38.479 *p* < 0.001**
Normal	2.51 ± 0.14	
Minor neurologic signs	2.29 ± 0.17	
Moderate neurologic signs	2.03 ± 0.13	
Severe neurologic signs	1.99 ± 0.36	

Mann Whitney *U* Test (*Z*); Kruskal Wallis *H* Test (*H*); Summary statistics are given as mean ± standard deviation and Median (minimum, maximum) values. Sections highlighted in bold are statistically significant (*p* < 0.05).

## Data Availability

The data presented in this study are available in article.
